# Mobile Microbiological Laboratory Support for Evaluation of a Meningitis Epidemic in Northern Benin

**DOI:** 10.1371/journal.pone.0068401

**Published:** 2013-07-02

**Authors:** Berthe-Marie Njanpop-Lafourcade, Stéphane Hugonnet, Honoré Djogbe, Agbenoko Kodjo, Adèle Kacou N’douba, Muhamed-Kheir Taha, Philippe Stoeckel, Bradford D. Gessner

**Affiliations:** 1 Agence de Médecine Préventive (AMP), Paris, France; 2 World Health Organization, Geneva, Switzerland; 3 Surveillance des Maladies, Ministry of Health, Cotonou, Bénin; 4 Regional Hospital Laboratory, Dapaong, Togo; 5 Pasteur Institute, Abidjan, Côte d’Ivoire; 6 Pasteur Institute, National Meningococcal Reference Center, World Health Organization Collaborating Center, Paris, France; 7 Agence de Médecine Préventive (AMP), Bureau de Liaison, Ferney-Voltaire, France; Health Protection Agency, United Kingdom

## Abstract

**Background:**

Fixed laboratory capacity in Africa may be inadequate; mobile microbiological laboratories may address this issue but their utility has seldom been evaluated.

**Methods:**

During 2012, the Benin Ministry of Health requested mobile microbiological laboratory (LaboMobil®) support following the failure of polysaccharide meningococcal A+C vaccine to prevent an epidemic in five Northern districts. Within four days, the intervention was initiated. A fixed site in Northern Togo, Pasteur Institutes in Côte d’Ivoire and France, and a research laboratory in Burkina Faso provided additional laboratory support.

**Results:**

Local laboratories initially reported most cases to have Gram-positive diplococci suggestive of pneumococcal meningitis. The LaboMobil® evaluated 200 cerebrospinal fluid (CSF) samples and 59 stored isolates collected from 149 individuals. Of the 74 individuals with etiologic confirmation, 60 (81%) had NmW135 and 11 (15%) NmX identified; no pneumococci were identified. Testing in France on 30 NmW135 and 3 NmX confirmed the etiology in all cases. All five districts had crossed the epidemic threshold (10 cases per 100,000 per week), all had NmW135 identified and four had NmX identified. NmX were identified as X:ST-181:ccST-181∶5-1∶10-1:F1–31 and NmW135 as W:ST-11: ccST-11∶5∶2:F1-1.

**Conclusions:**

In an area with limited local laboratory capacity, a mobile microbiology laboratory intervention occurred in four days through the cooperation of four African and one European country. Results were different from those reported by local laboratories. Despite the introduction of serogroup A meningococcal and 13-valent pneumococcal conjugate vaccines, endemic and epidemic meningitis will continue in the region, emphasizing the usefulness of the LaboMobil® in the short and medium term.

## Introduction

The African meningitis belt spans from Senegal in the West to Ethiopia in the East [1] and is characterized by seasonal endemic acute bacterial meningitis due to *Neisseria meningitidis* (Nm) and *Streptococcus pneumoniae* (Sp) [2], [3] as well as epidemic meningitis due to Nm, primarily serogroup A [4]. The introduction of MenAfriVac – a serogroup A conjugate vaccine – is likely to reduce NmA epidemics [5], [6], although definitive data on vaccine impact are lacking and questions remain regarding duration of immunity and optimal schedules. Introduction of pneumococcal conjugate vaccines (PCV) into routine infant immunization programs should further reduce meningitis incidence [7], although vaccine impact against serotype 1 (the most common serotype in the meningitis belt) remains undocumented.

Despite these advances, the meningitis belt is likely to continue to experience endemic and epidemic meningitis. NmX [8] and NmW135 [9] both can cause epidemic disease. Current PCVs cover a maximum of 13 serotypes and serotype replacement likely will occur, although the exact degree remains unknown [10]. Additionally, a high level of surveillance will be necessary to monitor the impact of new vaccines.

Unfortunately, a substantial portion of the meningitis belt population resides in rural areas with limited laboratory capacity [11]. The epidemiology of disease in these areas may differ from other areas due to differences in vaccine coverage, the prevalence of underlying illness and other risk factors for disease, climate, in- and out-migration, and other factors. Ideally, all sites would have high quality fixed laboratories, with an adequate number of trained human resources, appropriate equipment, and reliable stock of laboratory supplies. As an interim measure, the Agence de Médecine Préventive has developed a mobile microbiology laboratory (the LaboMobil®) to assist African partners with laboratory support for disease surveillance and response [12]. The current report describes an intervention conducted in Northern Benin in collaboration with the World Health Organization, the Ministry of Health of Benin, the AMP LaboMobil® based in Burkina Faso, the Pasteur Institute in Côte d’Ivoire, and an AMP and Ministry of Health supported research center in Dapaong, Togo.

## Methods

### Ethics Statement

This investigation was a public health response to an infectious disease emergency by the Benin Ministry of Health and organizations enlisted by the Ministry of Health to provide assistance. Generalizable knowledge was neither an a priori or a posteriori goal, other than the functioning of a piece of equipment, namely the LaboMobil®. Under these circumstances of public health non-research [13], institutional review board approval and individual informed consent were neither sought nor obtained. To protect the identity and privacy of individual patients, all data and specimens that were not in the control of the Benin Ministry of Health were de-identified by deleting name and date of birth and using a unique number for tracking; this process was used for specimens sent to Togo, Cote d’Ivoire, and France and the database used by AMP staff for analysis. All data were stored on password-protected servers or individual computers.

### LaboMobil® Description

**Figure 1:**
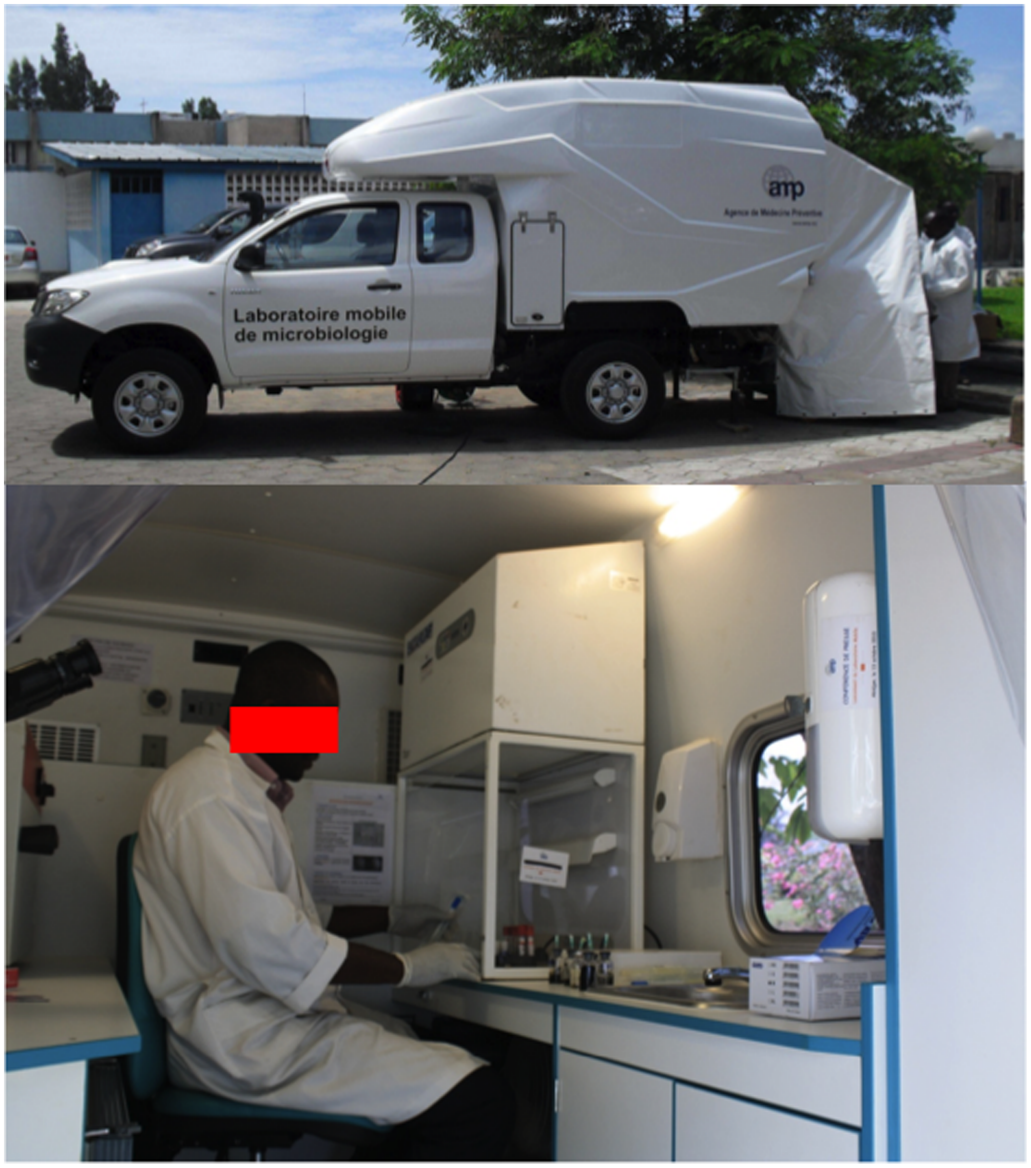
The exterior and interior of the LaboMobil®.

The LaboMobil® is a mobile microbiology laboratory first developed in 2003, and operational in Burkina Faso since 2005 and Côte d’Ivoire since 2010. The current third version is a Toyota Hilux WS 726 X-Tra Cabine 100 D40 4X pick-up with a Type II container and a heavy-duty shock-mounted chassis (Figure 1). This vehicle has a fully independent dual-chamber trailer laboratory, which includes air-conditioning, a HEPA Bio 99.9% air filter, and a negative pressure system. It is also equipped with stainless-steel bench tops, a mobile freezer unit, a refrigerator, a closed-circuit water system, an autoclave, a decontamination-resistant electrical system and furniture. The cabin is not removable, and a space between the cabin and the cell store generator contains the power cables and containers for petrol used to power the air-conditioning. The unit has an independent power supply with a 3 kW generator, HiPower batteries and a HiPower inverter as well as 220/12V power outlets in the external supply compartment.

### Context

Benin introduced into their routine Expanded Program on Immunization *Haemophilus influenzae* (Hi) type b containing pentavalent vaccine during June 2005, and 13-valent pneumococcal conjugate vaccine (PCV13) during July 2011. For the latter vaccine, coverage during 2011 was estimated as 36% and during 2012 as 98% (Benin Ministry of Health). Serogroup A meningococcal conjugate vaccine (MCV-A) was introduced in five meningitis belt departments in mass campaigns among persons age 1–29 years during November 2012, well after the current investigation (Meningitis Vaccine Project website. Available: http://www.meningvax.org/, last accessed May 15, 2013).

**Figure 2:**
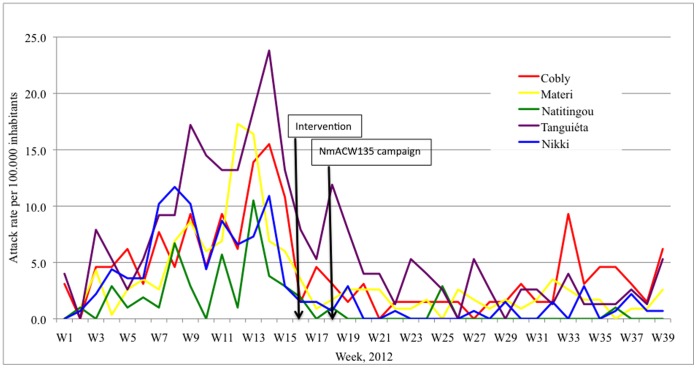
Attack rates of suspected acute bacterial meningitis reported to the Benin Ministry of Health from five districts, 2011/2012.

At the end of 2011, acute bacterial meningitis cases were reported from Northern Benin, which was followed in February 2012 by a mass immunization campaign using NmA+C polysaccharide vaccine among persons age one year and older. Despite this campaign, several areas continued to experience a large number of cases. Reports from the region indicated that some cases were due to serogroup W135 and during week 11, the Benin Ministry of Health requested an intervention with trivalent Nm A+C+W135 polysaccharide vaccine. Four districts in Atacora and one in Bourgou region passed the epidemic threshold (10 cases per 100,000 per week) including Nikki during week 7, Tanguiéta during week 9, Materi during week 12, Cobly and Natitingou during week 13 (Figure 2); the current investigation focused on these five districts.

**Figure 3:**
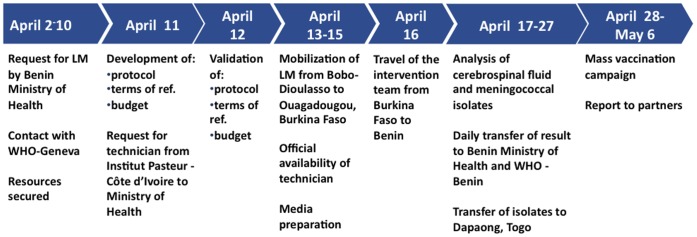
Timeline for the LaboMobil® (LM) intervention in Benin, April, 2012.

During week 14, the WHO and the Benin Ministry of Health requested the use of the LaboMobil® based in Burkina Faso to assist with the outbreak investigation (Figure 3). Securing adequate resources took 8 days, including 3 days for correspondence between AMP and WHO to define needed resources, 3 days for correspondence with the Cote d’Ivoire Ministry of Health through the Institut Pasteur, Cote d’Ivoire, and 2 days to agree on a budget. From the time adequate resources were secured, the mission was organized in four days, including a protocol, terms of reference, identification of human resources, delivery of laboratory reagents, and letter of authorization for a laboratory technician from the Pasteur Institute in Côte d’Ivoire. The mission lasted 15 days and was conducted during weeks 16 and 17. The LaboMobil® visited the districts of Tanguiéta, Natitingou, and Nikki and collected and analyzed specimens, transferred isolates to Dapaong, Togo for testing, and collected clinical and epidemiological data.

The district laboratory of Natitingou received cerebrospinal fluid (CSF) and isolates from the districts of Cobly, Materi, and Tanguiéta, as well as Natitingou itself. The three peripheral laboratories tested CSF with Gram stain and then sent the CSF to Natitingou, where culture was performed and isolates preserved in milk placed in a standard freezer of refrigerator. The Nikki laboratory also performed Gram stain and then transported CSF to the local reference laboratory in Parakou; while the LaboMobil® did not perform an intervention in Parakou, the team made a visit to retrieve specimens for Nikki.

### Laboratory Methods

The LaboMobil® team received fresh CSF from local clinicians and retrieved and tested stored CSF (if visibly cloudy) and stored isolates that had been collected previously. CSF and isolates were analyzed by classical microbiological methods for Gram stain and culture. CSF also underwent antigen detection via latex agglutination (Pastorex®, Biorad, Marne-la-Coquette, France) which detected Sp, Hib, and Nm A, C, and W135/Y. Isolates underwent meningococcal serogrouping using monovalent Difco™ Nm antiserum tests for serogroups A, C, W135, and X (Becton Dickinson Difco™ *Neisseria Meningitidis* Antisera package insert website. Available: http://www.bd.com/ds/technicalCenter/inserts/S1190_DifcoNeisseria(0703).pdf last accessed January 8, 2013). Stored isolates that could be revived and isolates from CSF were conserved for transportation in trans-Isolate [14] media or Amies (Portagerm®, Biomérieux, Marcy-l’Etoile, Lyon, France).

CSF and isolates were transported to several reference laboratories. These included the laboratory in Dapaong, Togo for antibiotic sensitivity testing, the Pasteur Institute in Côte d’Ivoire for polymerase chain reaction (PCR) analysis [15]–[17], and the French National Reference Center for Meningococci at the Pasteur Institute in Paris, France for quality control, phenotyping and sequence typing.

The Pasteur Institute in Côte d’Ivoire used a multiplex PCR technique which involved gene amplication of *crgA* for Nm, *bexA* for Hi, *lytA* for Sp, *siaD* for Nm genogroups B, C, Y, X, and W135, and *mynB* for NmA. The Pasteur Institute, Paris, France performed molecular analysis according to European Meningococcal Disease Society (EMGM) recommendations [18], [19] including PCR identification and genogrouping as well as testing for multilocus sequence typing (sequence type and clonal complex), PorA factor (PorA1(VR1), PorA2(VR2)) and FetA(Vr) on a selection of isolates as previously described [20].

## Results

The Benin Ministry of Health collected information on reported acute bacterial meningitis for the intervention districts from week 1 through week 26 of 2012. The peak weekly and cumulative attack rates per 100,000 population were, respectively, for Cobly 15.5 (week 14) and 125; for Materi 17.3 (week 12) and 112; for Natitingou 10.5 (week 13) and 46.8; for Nikki 11.7 (week 8) and 94.7; and for Tanguiéta 23.8 (week 14) and 211 (Figure 2).

The LaboMobil® evaluated 185 CSF samples and 59 isolates that had been collected and stored in Natitingou district laboratory before arrival as well as 15 CSF samples that were collected during the period the LaboMobil® was on site. Of the total 200 CSF specimens, the LaboMobil® staff performed latex agglutination on 40 and culture on 60 while the Pasteur Institute in Côte d’Ivoire performed PCR on 171 CSF specimens. Of the 59 stored isolates, LaboMobil® staff attempted to perform culture on all 59 but only 40 (68%) were viable. Of 11 NmX identified at the Natitingou Laboratory, LaboMobil® staff confirmed all 11 using monovalent antiserum; four of these 11 had associated CSF available with one having NmX confirmed by PCR. Of 21 NmW135 isolates stored at the Natitingou Laboratory that had associated CSF available, PCR testing confirmed 15 (71%) as NmW135. Nine stored CSF (three associated with NmX and six associated with NmW135) were negative by PCR.

The 200 CSF specimens and 59 stored isolates came from 149 individual patients, as some patients had up to four CSF samples collected. Among these 149 individual patients, an etiological agent was confirmed for 74 (50%) (Table 1). Of the 74 positive evaluations, NmW135 was identified for 60 (81%), NmX for 11 (15%), and Hi for 3 (4%). Of the 60 NmW135, 37 were positive by culture (5 from CSF and 32 from stored isolates), 17 by PCR on CSF, and 7 by latex agglutination on CSF (all the latter were confirmed by monovalent antisera). Of the 11 NmX, all were positive by culture (all from stored isolates). Two Hi were positive on PCR of CSF and one via latex agglutination of CSF. No pneumococci were identified.

**Table 1:** Findings on 149 patients with suspected acute bacterial meningitis, by district of residence and etiology, North Benin, weeks 9 through 17, 2012.

	Districts, with case counts (weekly incidence per 100,000 population)						
Etiology	Cobly	Materi	Natitingou	Nikki	Tanguiéta	Unknown	Total
NmW135*	4 (6.2)	23 (19.9)	8 (7.6)	3 (2.2)	22 (29.1)	–	60
NmX*	6 (9.3)	1 (0.9)	3 (2.9)	–	1 (1.3)	–	11
*H. influenzae*	–	–	–	–	3 (3.9)	–	3
Negative	19	16	1	–	30	2	68
Data not available on patient	–	–	–	–	5	2	7
Total known etiology	10 (15.5)	24 (20.8)	11 (10.5)	3 (2.2)	26 (34.3)	–	74
Total samples	29	40	12	3	61	4	149

* *Nm = Neisseria meningitidis*.

Four of the five districts (all except Nikki) were confirmed to be experiencing an epidemic during weeks 9 to 17, 2012 (Table 1). In all four districts, the epidemic was mixed NmW135 and NmX but predominantly NmW135. Confirmed cases occurred primarily among those <15 years of age (Table 2), who accounted for 60 of the 71 cases (85%) whose age and etiology were known.

**Table 2:** Findings on 149 patients with suspected acute bacterial meningitis, by age group (in years) and etiology, North Benin, weeks 9 through 17, 2012.

Test	<1 yr	1–4 yrs	5–14 yrs	15–29 yrs	30+ yrs	Unknown	Total
NmW135*	5	15	29	3	6	2	60
NmX*	1	4	3	2	–	1	11
*H. influenzae*	1	–	2	–	–	–	3
Negative	10	25	21	5	2	5	68
Data not available on patient	1	–	4	–	1	1	7
Total known etiology	7	19	34	5	6	3	74
Total	18	44	59	10	9	9	149

* *Nm = Neisseria meningitidis*.

Of the 48 total isolates (37 NmW135 and 11 NmX), 33 (30 NmW135 and 3 NmX) were tested at the Pasteur Institute, Paris, France and serogroups were confirmed for all 33. Genotyping documented that the three NmX were X :ST-181 :ccST-181∶5-1∶10-1 :F1-31; of 15 genotyped NmW135, all were W: ST-11: cc ST-11∶5∶2 :F1-1.

Of the 48 isolates, antibiotic sensitivity testing was performed in Dapaong, Togo for 19 NmW135 and 4 NmX; all were sensitive to ceftriaxone, chloramphenicol, and rifampicin. The Pasteur, Institute, Paris, France confirmed this sensitivity pattern for all 23 isolates.

One of the local district laboratories limited testing to Gram stain on CSF and results were compared to results from the LaboMobil® intervention using latex agglutination, culture, or PCR. This district laboratory reported 46 Gram-positive cocci via CSF microscopy of which LaboMobil® intervention identified 19 as NmW135, 3 as NmX, and 24 as negative. The same laboratory reported seven Gram-negative diplococci of which the LaboMobil® intervention identified six as NmW135 and one as negative. For four CSF samples, the laboratory identified no organisms while the LaboMobil® intervention identified two NmW135 and two Hi.

The LaboMobil® results confirmed the appropriateness of the earlier decision by the Benin Ministry of Health to implement Nm A+C+W135 polysaccharide vaccine mass campaigns, which was done during week 18. By the time vaccine arrived, all five evaluated districts had a weekly attack rate below 10 per 100,000 persons and thus were no longer considered to be experiencing an epidemic (Figure 2).

## Discussion

The current study involved an outbreak in Benin, the use of a mobile laboratory unit assigned to the Burkina Faso Ministry of Health, African reference laboratories in Togo and Côte d’Ivoire, and international support from AMP and the Pasteur Institute, Paris, France. Remarkably, the intervention was organized within four days of funding commitment. This level of regional collaboration would be impressive anywhere in the world. It was made possible by a common interest in identifying and limiting meningitis outbreaks as well as earlier efforts at cross-border cooperation by the World Health Organization (in Geneva and the African Regional and West African Sub-Regional Offices) as well as the West African Health Organization (West African Health Organization website. Available: http://www.wahooas.org/?lang=en, last accessed January 8, 2013).

The LaboMobil® intervention confirmed that multiple districts in North Benin were experiencing meningitis epidemics, that these outbreaks were due to NmW135 and to a lesser extent NmX, and that the affected population consisted mainly of persons age less than 15 years. At the start of the outbreak, during late 2011, immunization campaigns were initiated using polysaccharide NmA/C vaccine, which likely had little beneficial effect. Subsequent reports from the region before the arrival of the LaboMobil® indicated serogroup W135 was at least partly responsible for the epidemic. Conclusions were made more difficult by later results from a local laboratory identifying Gram-positive cocci, suggesting the presence of *Streptococcus pneumoniae*. Testing by the LaboMobil® and the associated reference laboratories in Natitingou and the Pasteur Institutes of Ivory Cost and France, however, confirmed the presence of NmW135 and NmX, supporting the earlier decision by the Ministry of Health to implement mass campaigns with W135-containing vaccine.

The cost per LaboMobil® unit varies depending on the specifications but is in the range of US $100,000–150,000. The total marginal cost of the current intervention – i.e., the cost given that a unit already existed in the region – was US $19,610, including US $6,510 for global human resources, US $7,220 for reagents, US $5,500 for supplies and per diem during the actual mission, and US $380 for shipping biological materials. These costs need to be compared to the costs of reinforcing local laboratories, recognizing that the LaboMobil® can travel to different sites as needed and the intervention itself will reinforce local laboratory capacity. Within the marginal costs, most would have occurred with a fixed laboratory evaluation, with the exception of the field supply and per diem costs. Lastly, some of the analyses we performed (such as etiological evaluation by PCR and genotyping) would be difficult to conduct in any scenario limited to reinforcing fixed rural African laboratories, and thus can be considered an added value of the mobile feature of the LaboMobil®.

While the LaboMobil® intervention occurred quickly and results were used rapidly to support the Ministry of Health’s decision on vaccine use, the vaccine intervention occurred late in the epidemic and thus its public health usefulness likely was limited, as suggested by the epidemic curve in the Figure. The usefulness of mobile laboratory interventions could be increased if they were fully integrated into Ministry of Health Infectious Disease Surveillance and Response systems, and thus available for deployment to rural areas at the start of an epidemic. However, even under these circumstances, difficulties remain. Outbreaks often are of short duration, particularly at the village or district level, and may be shorter for serogroup W135 than serogroup A. Additionally, insufficient W135 containing vaccine stocks are available, and none are routinely stored in affected countries.

The development of conjugate vaccines against meningococcal serogroup A and up to 13 pneumococcal serotypes likely will prove of great benefit to the inhabitants of the meningitis belt. However, this is not the end of the needed work. We and others have reported previously the existence of serogroup W135 [21] and X [8], [22], [23] epidemic and endemic disease. Additionally, serotype replacement may occur following PCV introduction and the meningitis belt has an unusual pneumococcal meningitis epidemiology with frequent disease throughout life, a predominance of serotype 1 after early childhood, many involved serotypes which can change even over a single year, and a seasonality mirroring that for meningococcus [2], [3], [24], [25]. These features emphasize the importance of continuing and strengthening surveillance, including for remote areas.

We support a long-term goal of adequate fixed laboratory and epidemiological capacity in all districts, which clearly offers the best chance of identifying and responding quickly to outbreaks. Unfortunately, many laboratories and surveillance systems in the meningitis belt suffer from insufficient funding, human resources, equipment, supplies, and training. For example, despite use of culture in Natitingou district laboratory, this laboratory did not have capacity to evaluate antibiotic susceptibility or have a system in place to ship biological samples to a reference laboratory. Moreover, in one of the largest district hospitals, care providers based case management on Gram stain results, which often were not correct.

Over the short- and medium-term, mobile laboratory units can play an important role by responding quickly to outbreaks. The delay of 15 days from request to first CSF analysis could be reduced substantially by local availability of a mobile laboratory and support staff. Mobile laboratories also can support studies by allowing the inclusion of rural populations, train local laboratory technicians, and support the rapid transport of specimens to local and international reference laboratories. These roles will be particularly important for surveillance that occurs in the context of new vaccine introductions, given the questions that remain about duration of protection, serogroup/type replacement, disease caused by etiologies not included in vaccines, geographic inequities in vaccine distribution, and other issues.
